# Contribution of CKD to mortality in middle-aged and elderly people with diabetes: the China Health and Retirement Longitudinal Study: CKD was a chronic stressor for diabetics

**DOI:** 10.1186/s13098-023-01083-0

**Published:** 2023-06-08

**Authors:** Xihong Liao, Ke Shi, Yumeng Zhang, Xiaoxu Huang, Ning Wang, Ling Zhang, Xiaohuan Zhao

**Affiliations:** 1 Department of Obstetrics and Gynecology, Shanghai Songjiang District Central Hospital, Shanghai, China; 2 Department of Ophthalmology, Shanghai General Hospital, Shanghai First People’s Hospital), Shanghai Jiao Tong University School of Medicine, Shanghai, China; 3 National Clinical Research Center for Eye Diseases, Shanghai, China; 4 Shanghai Key Laboratory of Fundus Diseases, Shanghai, China; 5 Department of Critical Care Medicine, Ruijin Hospital, Shanghai Jiao Tong University School of Medicine, Shanghai, China

**Keywords:** Diabetics, CKD, Mortality, CHARLS

## Abstract

**Background:**

The contribution of chronic kidney disease (CKD) to mortality in diabetic patients is unclear. This study aimed to explore the association between diabetics with CKD and mortality in middle-aged and elderly people of different ages.

**Methods:**

Data were obtained from the China Health and Retirement Longitudinal Study, including 1,715 diabetic individuals, 13.1% of whom also had CKD. Diabetes and CKD were assessed by combining the physical measurements and self-reports. We fitted Cox proportional hazards regression models to examine the effect of diabetics with CKD on mortality in middle-aged and elderly people. The risk factors for death were further predicted based on age stratification.

**Results:**

The mortality rate of diabetic patients with CKD (29.3%) was increased as compared to that of diabetic patients without CKD (12.4%). Diabetics with CKD were at a higher risk of all-cause mortality than those without CKD, with a hazard ratio of 1.921 (95% CI: 1.438, 2.566). Additionally, for participants 45 to 67 years of age, the hazard ratio was 2.530 (95% CI: 1.624, 3.943).

**Conclusions:**

Our findings suggested that, for diabetics, CKD was a chronic stressor that led to death in middle-aged and elderly people, especially among participants aged 45 to 67 years.

**Supplementary Information:**

The online version contains supplementary material available at 10.1186/s13098-023-01083-0.

## Background

Chronic kidney disease (CKD) is an abnormality of kidney function characterized by low estimated glomerular filtration rate (eGFR) based on serum creatinine measurements [1, 2]. Also, CKD is a risk multiplier in patients with hypertension and diabetes, suggested that CKD may have similar risk factors and share common pathophysiological factors with diabetics.

Some studies have investigated whether persons with CKD are at increased risk of death, particularly that caused by diabetes, but the results are inconsistent. In the Global Burden of Disease Study 2017, CKD accompanied with diabetes accounted for the largest share of absolute number of DALYs of any cause in 2017 [1]. The Australian diabetes, obesity and lifestyle study also found that diabetes and low eGFR were associated with increased all-cause mortality in Australian adults aged ≥ 25 years [3]. In a large multinational study of > 750,000 diabetics whose average age of 65, CKD was the most common concurrent cardiovascular disease and associated with increased mortality risks [4]. In type 2 diabetes, kidney disease-specific death rates vary greatly across studies. Nephropathy-specific mortality accounted for 11% of deaths in the WHO Multinational Study of Vascular Disease in Diabetes among those aged between 35 and 54 years at recruitment [5, 6] and only 0.9% of deaths in the Wisconsin Epidemiologic Study of Diabetic Retinopathy in participants of all ages [7]. There was no association between diabetes and end-stage renal disease (ESRD) events or death events in the Chronic Renal Insufficiency Cohort Study (CRIC) of people aged 60 [8]. The risk of death in people with diabetes and CKD varies greatly among studies. In addition, there is no further clarification of the differences in mortality risk for different age groups in the middle-aged and elderly population.

The China Health and Retirement Longitudinal Study (CHARLS) is a nationally representative longitudinal survey including assessments of diabetes, CKD, and the health circumstances of community residents. It provides an opportunity to examine the contribution of CKD to the mortality caused by diabetes in old people.

## Methods

### Study population

This study was based on CHARLS, a publicly used dataset containing a nationally representative sample of Chinese middle-aged and elderly community residents [9–11]. Specifically, CHARLS focuses on the health and retirement of middle-aged and elderly people in China, collecting information on a wide range of socioeconomic conditions and personal health conditions. Participants in the study covered 450 villages and urban communities in 28 provinces in China. The baseline survey of CHARLS was conducted from June 2011 to March 2012 and then followed up every 2 years. The data for this study included 7-year follow-up data from baseline. Finally, CHARLS has been approved by the Biomedical Ethics Review Committee of Peking University (IRB00001052-11015), and respondents were required to sign an informed consent form.

### Serum measurements

The serum measurement procedure has been published elsewhere [11]. After fasting overnight for at least 8 h, 8 ml fasting blood samples were collected in township hospitals or local Centers for Disease Control offices. Plasma was separated by centrifugation at 3200 rpm for 10–15 min within 1 h after collection, and kept in the dark at room temperature. Whole blood and centrifuged serum were stored at 4 ℃ in a local laboratory and were transported to the laboratory of Capital Medical University within 2 weeks at -80 ℃, where the blood glucose, blood lipids, glycosylated hemoglobin, and serum creatinine were measured.

### Diabetes

Diabetes was evaluated by combining the physical measurement and self-report. Based on current recommendations from the American Diabetes Association [12], diabetes was ascertained via (1) fasting plasma glucose ≥ 126 mg/dL, (2) random blood glucose ≥ 200 mg/dL, (3) glycated haemoglobin (HbA1c) ≥ 6.5%, or (4) a self-report of diabetes diagnosis by doctors or taking hypoglycemic drugs.

The onset age of participants with diabetes was also collected. For self-reported diabetes people, they were asked “When was diabetes first diagnosed or known by yourself?“ For those diagnosed with diabetes by physical measurement, their onset time was the time of blood glucose detection, that is, the baseline year 2011.

### Chronic kidney disease

CKD was defined as an eGFR of < 60 mL/min/1.73m^2^, as calculated using the CKD epidemiology collaboration (CKD-EPI) equations, or self-reported CKD [13].

The CKD-EPI equations was as follows:

eGFR (mL/min/1.73m^2^) = 135 × min(Cr/k, 1)^α^ × max(Cr/k, 1)^−0.601^ × min(Cys/0.8, 1)^−0.375^ × max(Cys/0.8, 1)^−0.711^ × 0.995^age^ ×0.969[if female]

Cr refers to serum creatinine (mg/dL), Cys refers to serum cystatin C (mg/liter), α is − 0.248 for females and − 0.207 for males, k is 0.7 for females and 0.9 for males.

### Mortality outcome

The CHARLS questionnaire followed the respondents to the 2011 baseline survey, with interviews in the next follow-up [14, 15]. At each follow-up interview, participants at baseline were recalled if possible. If a respondent’s death was reported, the team attempted to find a reliable informant so as to understand and determine the cause of death.

Mortality was determined by the interview status (alive or dead) of participants in waves 2, 3 and 4. The information of the interview date could be obtained from all three follow-ups, but the exact death time was only available in wave 2. If participants had survived during the observation period, the survival time was calculated as the interval between two surveys. If death events occurred, the survival time was the interval from the date of wave 1 to the date of participants’ death or the median time from the date of the first interview to the wave with death record.

### Covariates

To explain the confounding effect, we considered some covariates. According to the 2011 CHARLS questionnaire, we obtained some demographic information, including age, gender, education (primary or below, middle school, high school, or college or above) and marital status (married or partnered, otherwise), as well as lifestyle information, such as smoking status and drinking status (none, drink but less than once a month, or drink more than once a month).

Hypertension and dyslipidemia were defined by combining physical measurements and self-reports. Participants’ blood pressure was measured three times, and the average was taken as the final result. Hypertension was defined as systolic blood pressure ≥ 140 mmHg, diastolic blood pressure ≥ 90 mmHg, or a self-reported hypertension diagnosis by doctors or the taking of antihypertensive medicines [16]. Similarly, dyslipidemia was defined as the elevation of low-density lipoprotein cholesterol (≥ 4.14 mmol/L), triglycerides (≥ 2.26 mmol/L), and total cholesterol (≥ 6.22 mmol/L) or the decline of high-density lipoprotein level (< 1.04 mmol/L) or a self-reported dyslipidemia diagnosis by doctors [17].

### Statistical analysis

In our study, diabetics with or without CKD were the primary exposure of interest, while other independent variables served as covariates. Continuous variables were shown as means ± standard deviations (SDs), and categorical variables were presented as numbers and percentages. To compare the baseline characteristics of diabetic patients with or without CKD, we used analysis of variance and Chi-square test according to the data type and distribution. The overall significance of univariate survival analysis was determined by a log-rank test using Kaplan-Meier analysis. Cox proportional-hazards regression was used to evaluate hazard ratios (HRs) for all-cause mortality. Covariates and CKD status were included in the Cox proportional-hazards regression models. Because the risk of mortality was strongly associated with increasing age, age was considered as the time scale in the Cox proportional-hazards regression, allowing the models to compare risk for people of comparable ages. The HR value was calculated for each variable, including CKD, and the statistical significance of the interaction term in the regression analysis was determined by generating time-related covariates for each variable from the interaction between the variable and the logarithm of follow-up time. Analyses were divided into two age groups according to the mean age at death (ages 45 to 67 years and ages 68 + years). *P*-values less than 0.05 were considered statistically significant.

## Results

There were 17,708 participants at baseline, of which 2,205 had diabetes. Some individuals with missing values were discarded, and a total of 1,715 individuals were included in our study. Among these, 225 (13.1%) also had CKD. Those with CKD were more likely to be smokers and have hypertension and dyslipidemia (Table 1).

**Table 1:** Participants Characteristics for study population

Characteristics at Baseline Examination	Diabetics without CKD (n = 1490)	Diabetics with CKD (n = 225)	*P*
Onset age (yrs)	58.59(9.8)	61.60(10.5)	< 0.001^b^
Male gender	47.2%(703)	50.2%(113)	0.395^a^
Education			0.613^a^
Primary or below	68.0%(1013)	72.4%(163)	
Middle school	20.1%(299)	17.3%(39)	
High school	6.6%(99)	5.8%(13)	
College or above	5.3%(79)	4.4%(10)	
Marital status: Married or partnered	89.2%(1329)	86.2%(194)	0.187^a^
Smoking	38.5%(573)	45.8%(103)	0.036^a^
Drinking			0.093^a^
Drink more than once a month	24.3%(362)	19.6%(44)	
Drink but less than once a month	6.5%(97)	5.8%(13)	
None	69.2%(1031)	74.7%(168)	
Hypertension	39.7%(592)	50.2%(113)	0.003^a^
Dyslipidemia	18.4%(274)	30.2%(68)	< 0.001^a^

^a^ Cochran-Mantel-Haenszel χ^2^ test

^b^ One-way analysis of variance

Abbreviations: CKD, chronic kidney disease

**Fig. 1:**
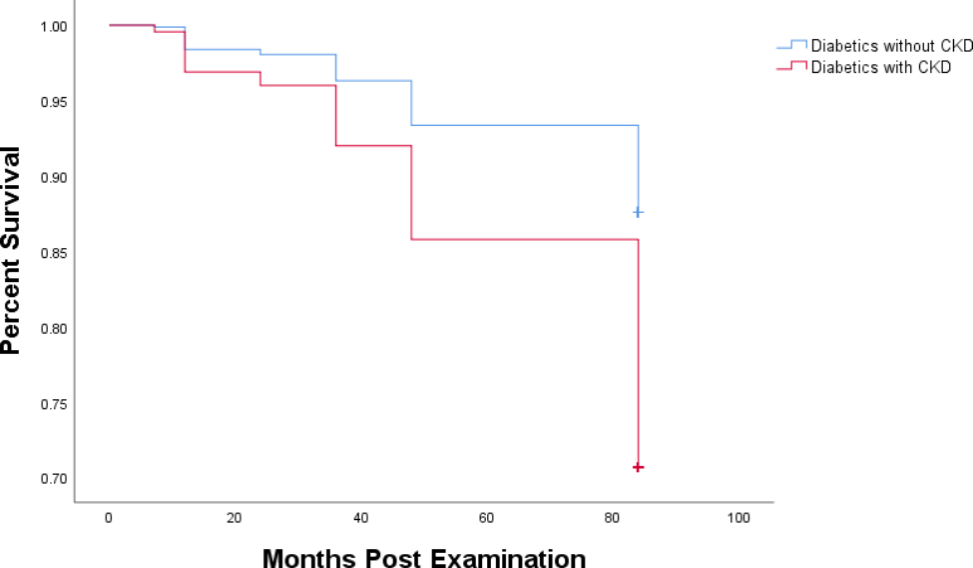
Kaplan-Meier plot showing diabetes-related mortality rates by CKD status

In this analysis, we examined 7-year all-cause mortality from the 2011 baseline to the 2018 interview. During these 7 years, 251 (14.6%) diabetic participants died; among them, 66 (26.3%) also had CKD. Individuals with CKD were significantly more likely to die as compared with their peers without CKD of comparable age (Fig. 1). The mortality rate among diabetic patients with CKD was 29.3%, as compared with 12.4% for those without CKD (log rank test p < 0.001.) After a closer consideration of the proportional-hazards hypothesis, it became clear that this association was driven by data from older people in the distribution and that, for statistical reasons, the cohort needed to be age-stratified. Supplemental Figs. 1 and 2 show mortality based on CKD status during the 7-year follow-up period after age stratification and adjustment for age and gender. Whether participants were 45 to 67 years of age (Supplemental Fig. 1; log rank test p = 0.001) or over 68 years old (Supplemental Fig. 2; log rank test p < 0.001), the mortality of people with CKD was higher than that of people without CKD.

Across all participating populations, a total of 251 participants died. The characteristics for those who died are provided in terms of CKD and stratified by age group in Table 2. There were 1,306 diabetic participants 45 to 67 years of age at their baseline examination, of whom 66 (5.1%) died; among these, 15 had CKD, and 51 had no CKD. Of the 409 individuals over 68 years old, 185 (45.2%) died; among these, 51 had CKD, and 134 had no CKD. Among those who died at 45 to 67 years of age, persons who died with CKD were significantly more likely to have dyslipidemia than persons without CKD.

**Table 2:** Baseline characteristics by CKD in those who died

Characteristics at Baseline Examination	Participants 45 to 67 Years of Age (n = 66)			Participants 68 + Years of Age (n = 185)		
	Diabetics without CKD (n = 51)	Diabetics with CKD (n = 15)	*P*	Diabetics without CKD (n = 134)	Diabetics with CKD (n = 51)	*P*
Onset age (yrs)	65.25(12.3)	69.67(6.1)	0.184	65.66(11.1)	64.94(13.0)	0.706
Male gender	52.9%(27)	66.7%(10)	0.346	56.0%(75)	56.9%(29)	0.913
Education			0.763			0.932
Primary or below*	74.5%(38)	73.3%(11)		81.3%(109)	80.4%(41)	
Middle school	21.6%(11)	20.0%(3)		12.7%(17)	11.8%(6)	
High school	2.0%(1)	0.0%(0)		2.2%(3)	2.0%(1)	
College or above	2.0%(1)	6.7%(1)		3.7%(5)	5.9%(3)	
Marital status: Married or partnered	76.5%(39)	73.3%(11)	0.803	80.6%(108)	74.5%(38)	0.364
smoking	39.2%(20)	40.0%(6)	0.956	50.0%(67)	51.0%(26)	0.905
drinking			0.821			0.893
Drink more than once a month	15.7%(8)	20.0%(3)		20.1%(27)	21.6%(11)	
Drink but less than once a month	3.9%(2)	6.7%(1)		4.5%(6)	5.9%(3)	
None	80.4%(41)	73.3%(11)		75.4%(101)	72.5%(37)	
Hypertension	58.8%(30)	80.0%(12)	0.134	47.0%(63)	54.9%(28)	0.338
Dyslipidemia	11.8%(6)	40.0%(6)	0.013	16.4%(22)	23.5%(12)	0.264

Abbreviations: CKD, chronic kidney disease

To further clarify the factors affecting the survival time of diabetic patients, we constructed a Cox proportional-hazards regression model (Table 3). The factors predictive of mortality included CKD (HR = 1.921, 95% CI: 1.438–2.566), onset age (HR = 1.058, 95% CI: 1.043–1.072), male (HR = 1.504, 95% CI: 1.064–2.125), and hypertension (HR = 1.520, 95% CI: 1.174–1.969). Compared to single status, married or partnered status predicted a lower risk of death (HR = 0.718, 95% CI: 0.519–0.994).

**Table 3:** Cox Proportional Hazards Regression Models of CKD

Variable	HR	95% CI	*P*
Chronic kidney disease: Yes vs. No	1.921	1.438, 2.566	< 0.001
Onset age per year	1.058	1.043, 1.072	< 0.001
Sex: Male vs. Female	1.504	1.064, 2.125	0.021
Education: Primary or below vs. College or above	1.437	0.751, 2.747	0.273
Education: Middle school vs. College or above	1.393	0.688, 2.820	0.358
Education: High school vs. College or above	0.622	0.211, 1.838	0.391
Marital status: Married or partnered vs. Otherwise	0.718	0.519, 0.994	0.046
Smoking: Yes vs. No	1.316	0.946, 1.831	0.103
Drinking: Drink but less than once a month vs. Drink more than once a month	0.941	0.499, 1.773	0.850
Drinking: None vs. Drink more than once a month	1.581	1.121, 2.228	0.009
Hypertension: Yes vs. No	1.520	1.174, 1.969	0.002
Dyslipidemia: Yes vs. No	0.863	0.617, 1.207	0.389

Abbreviations: HR, hazard ratio; CI, confidence interval

We further predicted the risk factors for death based on age stratification (Table 4). In participants 45 to 67 years of age, CKD, male gender, being single, and hypertension were considered factors predictive of mortality after controlling for potential confounders. In the older group, factors predictive of mortality included CKD, smoking, and hypertension after adjusting for age and concomitant conditions.

**Table 4:** Cox Proportional Hazards Regression Models of CKD in different age groups

Variable	Participants 45 to 67 Years of Age (n = 1306)		Participants 68 + Years of Age (n = 409)	
	HR(95%CI)	*P*	HR(95%CI)	*P*
Chronic kidney disease: Yes vs. No	2.530(1.624, 3.943)	< 0.001	1.591(1.087, 2.329)	0.017
Sex: Male vs. Female	2.193(1.293, 3.717)	0.004	1.092(0.695, 1.715)	0.702
Education: Primary or below vs. College or above	1.438(0.575, 3.598)	0.438	1.994(0.795, 5.000)	0.141
Education: Middle school vs. College or above	1.309(0.501, 3.420)	0.582	1.239(0.419, 3.660)	0.698
Education: High school vs. College or above	0.508(0.136, 1.899)	0.314	0.705(0.081, 6.127)	0.751
Marital status: Married or partnered vs. Otherwise	0.497(0.288,0.859)	0.012	0.776(0.528, 1.142)	0.199
Smoking: Yes vs. No	1.045(0.640, 1.709)	0.859	1.602(1.039, 2.471)	0.033
Drinking: Drink but less than once a month vs. Drink more than once a month	1.007(0.440, 2.301)	0.987	1.027(0.379, 2.781)	0.959
Drinking: None vs. Drink more than once a month	1.246(0.778, 1.996)	0.361	2.053(1.210, 3.483)	0.008
Hypertension: Yes vs. No	1.652(1.128, 2.419)	0.010	1.493(1.047, 2.128)	0.027
Dyslipidemia: Yes vs. No	0.814(0.509, 1.301)	0.390	0.780(0.481, 1.266)	0.315

Abbreviations: HR, hazard ratio; CI, confidence interval

## Discussion

In the middle-aged and elderly population, diabetic patients with CKD have an increased risk of death after considering lifestyle and chronic systemic diseases. In particular, CKD has a greater impact on the risk of death among diabetic participants aged 45 to 67 than those aged 68 or above. We speculate that, with older age, the impact of physiological aging on death increases, which may reduce the contribution of any specific disease, such as CKD, to the risk of death.

Diabetes with CKD include the following three cases: CKD caused by diabetes mellitus (diabetic kidney disease, DKD), diabetes mellitus combined with CKD but without a causal relationship (nondiabetic kidney disease, NDKD), and a combination of DKD and NDKD [18]. The mechanisms underlying the relationship between diabetics with CKD and mortality are still not fully understood. We speculated that diabetics with CKD were more likely to die for the following reasons. First of all, due to the decline in GFR and the abilities of synthetic renal hormones, diabetics with CKD usually develop metabolic abnormalities, including hyperphosphatemia, secondary hyperparathyroidism, hyperkalemia, and metabolic acidosis, which would increase the risk of death. Secondly, diabetes with CKD is also associated with increased inflammatory factors, abnormal apolipoprotein levels, elevated plasma homocysteine levels, enhanced coagulation, anemia, left ventricular hypertrophy, increased arterial calcification and endothelial dysfunction [19–21]. Whether and how these and other factors interact to increase the risk of death remains unclear, but it is the focus of ongoing investigations. In addition, the risk and severity of other diabetic complications were increased in diabetics with CKD, including retinopathy, neuropathy, gastroparesis, sexual dysfunction, cognitive impairment, sleep and mood disorders, heart failure, atrial fibrillation, and cardiovascular and foot diseases [21]. The presence of CKD in patients with diabetes could be regarded as a risk marker for each of these situations, but it is usually an aggravating factor [22]. The more severe the impairment of renal function, the greater the risk of cardiovascular and other complications. Studies have shown that the incidence of myocardial infarction and stroke in patients with diabetes and CKD is about two times that of patients with diabetes but without kidney disease [23, 24].

Our results were supported by some previous studies [1, 3, 4, 25]. In the Global Burden of Disease Study 2017, CKD ranked as the twelfth leading cause of death in 2017, and CKD due to diabetes accounted for 30.7% of CKD DALYs. Diabetes and low eGFR were strongly associated with increased all-cause mortality in the Australian Diabetes, Obesity, and Lifestyle study. In a large multinational study of > 750,000 diabetics, 6.48% had manifestations of CKD, which were associated with an increased risk of all-cause death. In a cohort of 12,570 diabetic patients involving seven Veterans Affairs hospitals, the mortality rate among diabetic patients with CKD was 20.1 deaths/100 person-years, as compared with 4.7 deaths/100 person-years among diabetic patients not affected by CKD [25]. The current study supports the association between CKD and mortality and extends the findings regarding older Chinese diabetics with CKD and their increased death risk.

However, CRIC Study found no association between diabetes and end-stage renal disease (ESRD) events or death events in 1,798 participants with eGFR < 30 ml/min/1.73 m^2^ [8]. The eGFR of the enrolled patients was < 30 ml/ min/1.73 m^2^ with “severely decreased” kidney function (CKD stage G4) [26, 27], which may be why these results are inconsistent with the current results.

In our study, the risk of death increased by 1.058 times for every year of onset age in the middle-aged and elderly population. Both males and single individuals increase the risk of death, with males having a 1.504 times higher risk of death compared to female participants. Married or partnered individuals were associated with a lower risk of death as compared with unmarried ones, which can be explain by their improved health habits and decreased psychiatric symptomatology and syndromes [28–31]. In addition, underlying diseases, such as high blood pressure, are also important factors that increase the risk of death, which is also consistent with previous findings [32, 33]. Therefore, aggressive blood pressure control will reduce the risk of death.

In addition, we found that factors affecting mortality risk vary by age group. For participants 45 to 67 years of age, men were at a higher risk of death than women, which was consistent with previous studies [34–36]. Gender differences in psychosocial and environmental exposure are believed to be responsible for the difference in mortality between men and women. For participants older than 68 years old, smoking increases the risk of death, and the risk of death among smokers is 1.602 times that of non-smokers, which suggests that, for elderly people, smoking cessation is one of the most effective interventions to reduce the risk of death.

Patients with hypertension were at higher risk of death than those without hypertension, especially for participants aged 45–67 years. In the Trials of Hypertension Prevention Study, a direct linear association between average sodium intake and mortality was found in adults 30 to 54 years of age [37]. In a West Jerusalem longitudinal prospective cohort study, increased systolic blood pressure was not associated with increased 5-year mortality among a representative cohort of community-dwelling 85-year-olds [38]. The strong survival bias may be one of many potential explanations: healthy survivors reach old age, while susceptible subjects die at a younger age.

Based on our results, in addition to glycemic control, early interventions for other comorbidities, especially CKD and hypertension, are also required for diabetic patients. At the same time, attention should be paid to lifestyle interventions, including smoking cessation. In addition, it also should be paid attention to the association between diabetes in pregnancy, diabetes retinopathy, acute and critical diabetes and mortality. Diabetes in pregnancy increased the risk of both perinatal and postneonatal death for hyperglycaemia’s fetal toxicity [39]. Diabetes retinopathy was predictive of all-cause mortality in diabetes [40]. Diabetic ketoacidosis, hyperglycaemic hyperosmolar state and hypoglycaemia are serious complications of diabetes mellitus that are highly associated with increased mortality [41].

A major strength of this study was its population-based design for a nationally representative high-risk population aged ≥ 45 years old, allowing for reasonable conclusions. Furthermore, our study provides relatively strong evidence by using a large sample size for blood tests and physical measurements. In addition, a wide range of covariates, such as hypertension, dyslipidemia, smoking status, and drinking status, were considered in our study. Some limitations of our study must also be considered. First, CHALRS does not collect information on certain confounding factors, such as family history, CKD-related complications, and other chronic diseases. In addition, due to data limitations, CKD staging cannot be considered in this study, so the results should be interpreted with caution. Moreover, we did not analyze the cause of death of CKD with diabetes due to the lack of data. Despite these limitations, the strengths of this study include a nationally representative and population-based sample, a 7-year follow-up, a stratified analysis of age groups, and strict quality control measures for the data.

## Conclusions

To conclude, we identified diabetes patients with CKD as having an increased risk of death among middle-aged and elderly Chinese people. In particular, CKD has a great impact on the risk of death among diabetic participants aged 45 to 67 years. At the same time, the risk of death is strongly related to age, smoking, and hypertension, which should be considered when designing and evaluating CKD treatment and solving patient management problems.

## Electronic supplementary material

Below is the link to the electronic supplementary material.

Supplemental Figure 1: Kaplan-Meier plot showing diabetes-related mortality rates by CKD status in 45 to 67 years of age participants

Supplemental Figure 2: Kaplan-Meier plot showing diabetes-related mortality rates by CKD status in 68+ years of age participants

## Data Availability

No additional data available.

## List of Abbreviations

CHARLS: The China Health and Retirement Longitudinal Study
CKD: Chronic kidney disease
CKD-EPI: CKD epidemiology collaboration
CRIC: The Chronic Renal Insufficiency Cohort Study
DKD: Diabetic kidney disease
NDKD: Nondiabetic kidney disease
eGFR: Estimated glomerular filtration rate
ESRD: End-stage renal disease
HR: Hazard ratios
