# Supplementary figures and images for: Contribution of CKD to mortality in middle-aged and elderly people with diabetes: the China Health and Retirement Longitudinal Study: CKD was a chronic stressor for diabetics
Source: Diabetol Metab Syndr. 2023 Jun 8;15:122. doi: 10.1186/s13098-023-01083-0 (PMC10249178; doi:10.1186/s13098-023-01083-0)

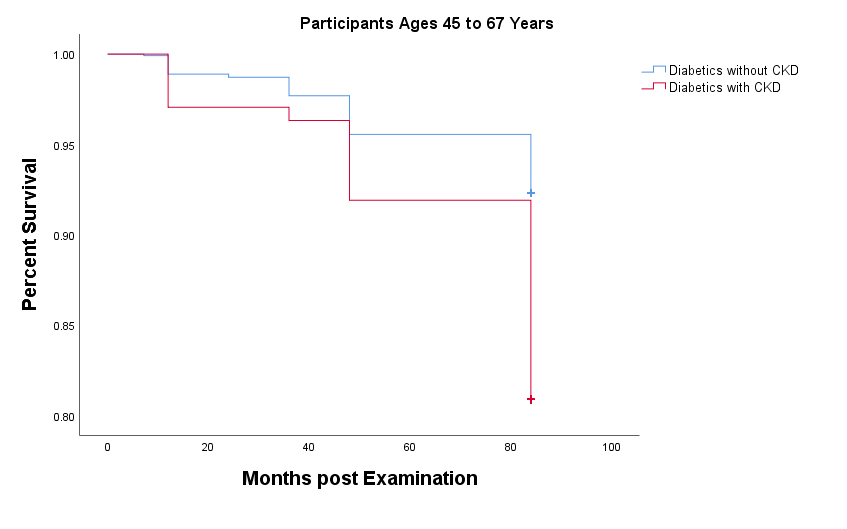

Supplement: Supplementary file 1 — Supplemental Figure 1: Kaplan-Meier plot showing diabetes-related mortality rates by CKD status in 45 to 67 years of age participants [file 13098_2023_1083_MOESM1_ESM.png]

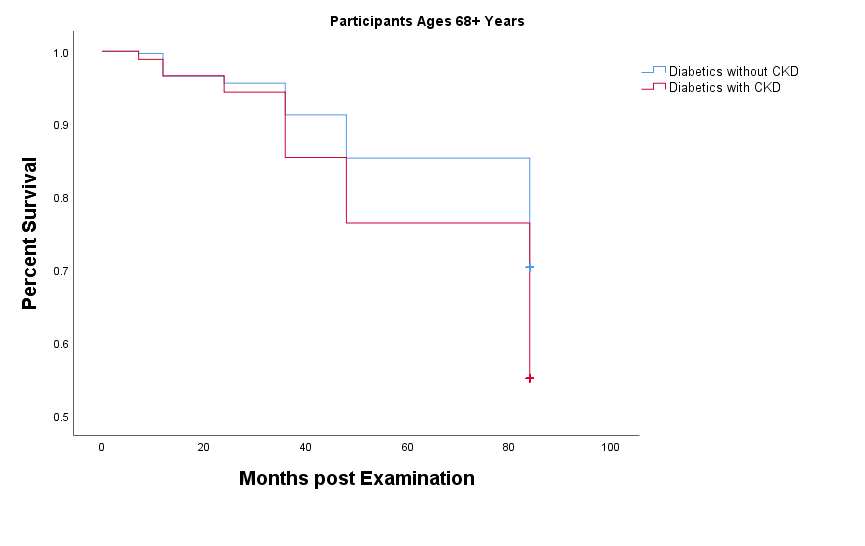

Supplement: Supplementary file 2 — Supplemental Figure 2: Kaplan-Meier plot showing diabetes-related mortality rates by CKD status in 68+ years of age participants [file 13098_2023_1083_MOESM2_ESM.png]
